# Compliance with the EAT-Lancet diet and risk of colorectal cancer: a prospective cohort study in 98,415 American adults

**DOI:** 10.3389/fnut.2023.1264178

**Published:** 2023-10-19

**Authors:** Xiaorui Ren, Chuanchuan Yu, Linglong Peng, Haitao Gu, Yi Xiao, Yunhao Tang, Hongmei He, Ling Xiang, Yaxu Wang, Yahui Jiang

**Affiliations:** ^1^Department of Gastrointestinal Surgery, The Second Affiliated Hospital of Chongqing Medical University, Chongqing, China; ^2^Department of Medical Statistics, School of Public Health, Sun Yat-sen University, Guangzhou, Guangdong, China; ^3^Department of Clinical Nutrition, The Second Affiliated Hospital of Chongqing Medical University, Chongqing, China

**Keywords:** EAT-lancet diet, colorectal cancer, cox hazards regression analysis, prostate, lung, colorectal, ovarian cancer screening trial

## Abstract

**Background:**

The EAT-Lancet diet (ELD) is a recommended dietary pattern for achieving simultaneous improvements in both individual health and environmental sustainability. While research on the association between ELD and colorectal cancer (CRC) remains scarce, the potential impact of nutrition on CRC prevention and progression is a topic of growing interest. This study aims to investigate the relationship between adherence to the ELD and the risk of CRC, shedding light on the role of nutrition in CRC prevention.

**Methods:**

A total of 98,415 participants were included. A Diet History Questionnaire (DHQ) was used to collect dietary information, and an ELD score was used to assess adherence to ELD. Higher scores indicated greater adherence. Cox hazard regression analyses were conducted to examine whether there were associations between the ELD score and CRC risk. The restricted cubic spline (RCS) model was used to further explore the dose-response association between the ELD score and CRC incidence. Subgroup analyses were conducted to identify potential modifiers that interacted with ELD on CRC incidence, and sensitivity analyses were performed to evaluate the robustness of the established association.

**Results:**

During a mean follow-up of 8.82 years, a total of 1,054 CRC cases were documented. We found a statistically significant correlation between the ELD score and CRC risk (Q4 vs. Q1: HR 0.81, 95% CI 0.67–0.98; P for trend = 0.034) after adjusting for potential confounders. No statistically significant associations were discovered between ELD adherence and CRC by anatomical site. Subgroup analyses found no interactional factor, sensitivity analyses, and the RCS model showed a robustness and linearity association (P-linearity >0.05).

**Conclusion:**

We concluded that adherence to ELD contributes to the prevention of CRC.

## 1. Introduction

In the United States, colorectal cancer (CRC) is an important cause of cancer burden. It is reported to be the third most commonly diagnosed cancer and the third cause of cancer-associated death in both men and women (1). In 2023, it is estimated to have 153,020 new cases of CRC and 52,550 CRC deaths in the United States (1). The occurrence and development of CRC is a slow and long-term process, which provides opportunities for some preventive measures (2). In Western countries, diet is one of the most important risk factors for CRC, making it a possible preventive target (1).

Epidemiological research suggests that many foods can reduce the risk of CRC (3–6). In a prospective study of UK Biobank, participants eating more red and processed meat had a higher risk of CRC (3), while a meta-analysis by Schwingshackl et al. (4) found an inverse association between vegetables, fruit, and whole grains and CRC. A systematic review showed a protective effect of fish on CRC (5), while a study in two prospective US cohorts found an adverse effect of added sugars (6). These studies focused on specific foods and thus may not offer a comprehensive understanding of an ideal diet for overall health. Dietary patterns, which characterize a variety of foods, nutrients, and beverages, may serve as useful tools to represent the overall effects of diet on the risk of health outcomes.

Recently, the EAT-Lancet diet (ELD) was introduced as a scientifically optimized diet for nutrition and certain environmental indicators (7). In 2019, the EAT–Lancet Commission, made up of experts from diverse fields such as human health, agriculture, political science, and environmental sustainability, proposed for the first time the ELD, which is universally applicable to all food cultures and production systems in the world with high potential for local adaptation and scalability (7). Further research showed that ELD is affordable in most countries, including the United States (8). The ELD encourages the intake of vegetables, fruits, whole grains, legumes, nuts, unsaturated oils, and fish while limiting the intake of beef, lamb, pork, poultry, eggs, dairy products, potatoes, and added sugars. The dietary components of ELD are similar to those of the Mediterranean diet (MD) (high intakes of vegetables (excluding potatoes), fruits, whole grains, legumes, nuts, and fish, while a low intake of red and processed meats) (9), which has been widely recognized for its health benefits (10–12). Compared to MD, ELD is more environmentally friendly and requires less water (13). Adherence to ELD may greatly benefit human health. For example, adherence to ELD could reduce annual mortality by 19.0–23.6% (14) and could also reduce the risk of cardiovascular disease and diabetes (15–17). However, Berthy et al. (18) comprehensively analyzed the association between ELD, cardiovascular disease (CVD), and cancer risk. They concluded that adherence to the ELD could decrease the risk of cancer only in some subgroups but found no association with CVD risk.

To date, research focused on the ELD and CRC risks is scarce. Therefore, we conducted this analysis to explore the relationship between ELD adherence and CRC risk in 98,415 subjects aged 55 to 74 years from the Prostate, Lung, Colorectal, and Ovarian (PLCO) cohort.

## 2. Methods

### 2.1. Study population

The PLCO Cancer Screening Trial is a large multicenter randomized controlled trial designed to evaluate the effectiveness of screening methods for prostate, lung, colorectal, and ovarian cancer. More information about the PLCO Cancer Screening Trial has been described elsewhere (19). In 10 selected screening centers nationwide in the United States, 154,887 men and women aged 55 to 74 years were enrolled in the PLCO cancer screening trial between 1993 and 2001 and then randomized to control or intervention arms in a 1:1 ratio (control arm received usual care, while intervention arm received additional screening care) (20). At baseline, participants were administered some self-reported questionnaires, such as the Baseline Questionnaire (BQ), Supplementary Questionnaire (SQX), and Diet History Questionnaire (DHQ), to collect individual characteristics, including diet and other cancer risk factors. All screening procedures and individual medical record abstracting were performed by trained and certified specialists, and the cause of death was certified by the Death Review Committee (DRC) (19, 21). The PLCO Cancer Screening Trial was approved by the National Cancer Institute (NCI), one of the components of the National Institutes of Health (NIH) (20), and each of the 10 screening centers involved in the study, all participants provided explicit, informed, and written consent. Our research was carried out with the approval of the NCI (project number: PLCO-1231).

**Figure 1:**
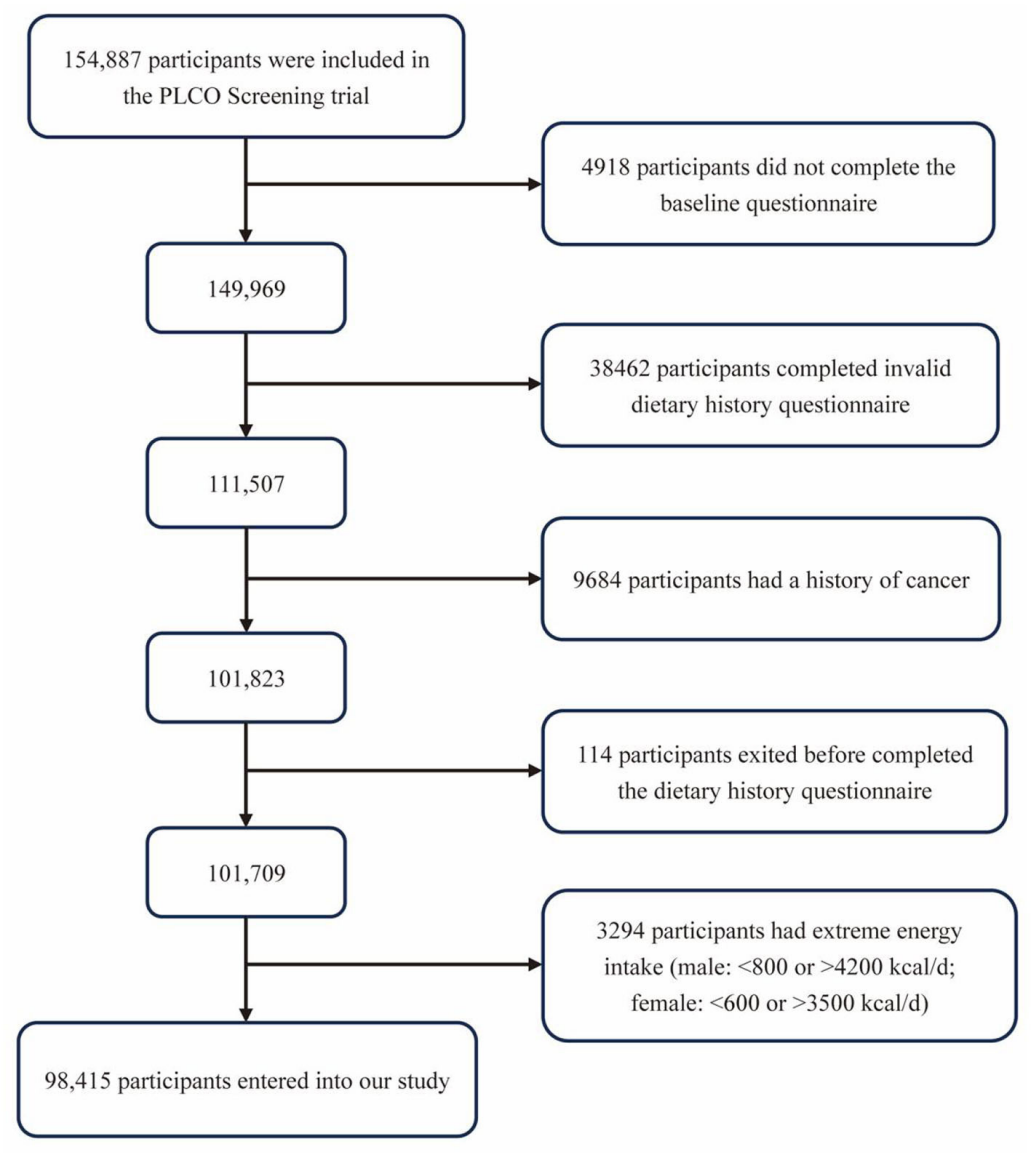
The flowchart for identifying eligible subjects. PLCO, Prostate, Lung, Colorectal, and Ovarian.

In consideration of the objective of our study, we further excluded subjects as follows: (1) did not complete the BQ (*n* = 4,918); (2) did not complete a valid DHQ (valid DHQ refers to DHQ with date of completion, <8 missing frequency responses, still alive when completed DHQ, and participants with no extreme calorie intake, which means participants in the first or last percentile by gender) (*n* = 38,462); (3) had a history of any cancer (except non-melanoma skin cancer) (*n* = 9,684); (4) exited before accomplishing the DHQ (*n* = 114); (5) had unbelievable energy intake unbelievable energy intake refers to food energy intake from a diet <800 kcal or >4200 kcal for men and <600 kcal or >3500 kcal for women (22) (*n* = 3,294). Finally, 98,415 participants were included in our analyses (Figure 1).

### 2.2. EAT-lancet diet compliance

Dietary information was collected by the above-mentioned DHQ, a food frequency questionnaire (FFQ) developed by members of the Risk Factor Monitoring and Methods Branch (RFMMB). The DHQ consists of 124 food items, includes portion size and dietary supplement questions, and provides reliable estimates of nutrient intake. The validity and reliability of DHQ have been tested elsewhere (23).

Compliance with ELD was assessed using the ELD scores, which are derived from the study of Stubbendorff et al. (24), who established ELD scores based on the recommendation of the EAT-Lancet Commission. In their research, a total of 14 food components were described as “emphasized foods” or “limited foods.” Emphasized food components included whole grains, vegetables (except starch vegetables), fruits, legumes, nuts, unsaturated oils, and fish, while limited food components consisted of potatoes, dairy, eggs, poultry, pork, beef, lamb, and added sugar. Food components were described in grams per day and were dealt with based on an energy intake of 2500 kcal, consistent with the dietary target intake recommended by the EAT-Lancet Commission (7). According to the quantity of individual food intake, each component ranged from 0 to 3 points, with a possible total score of 0 to 42 (Supplementary Table 1). Specifically, emphasized food groups were given positive scores, while limited food groups were given negative scores. In other words, 3 points indicated high compliance, and 0 points indicated low compliance. Higher scores indicated greater adherence to ELD.

### 2.3. Assessment of covariates

The BQ, DHQ, and SQX were used to collect information involving demographic and lifestyle factors, such as age, sex, race, body mass index (BMI), smoking status, pack-year of smoking, drinking status, physical activity level, aspirin or ibuprofen consumption, family history of CRC, history of diverticulitis, history of colorectal polyps, history of colon comorbidities (including ulcerative colitis, Crohn's disease, Gardner's syndrome, or familial polyposis), energy intake from diet, protein intake from diet, carbohydrate intake from diet, and fat intake from diet. Diet-associated covariates, such as energy intake from diet, protein intake from diet, carbohydrate intake from diet, and fat intake from diet, were collected by DHQ, physical activity level was derived from SQX, and all the other covariates were taken from BQ. Race was classified as white or non-white. BMI was calculated as weight in kilograms divided by height in meters squared. Smoking status was described as non-smokers and previous/current smokers. Physical activity level was calculated as the sum of self-reported minutes of moderate to vigorous activity in a week.

### 2.4. Ascertainment of outcomes

In this study, the primary outcome was the diagnosis of CRC. CRC was defined based on the definitions by the International Classification of Diseases for Oncology (ICD-O-2; codes: proximal colon cancer: C180-C185, distal colon cancer: C186-C187, and rectal cancer: C19-C20) (25). Proximal colon cancer includes cecum, appendix, ascending colon, hepatic flexure, transverse colon, and splenic flexure colon cancer. Distal colon cancer includes descending and sigmoid colon cancer (26). Participants were sent a self-reporting annual study update form to report any new CRC diagnoses they received, including the date and type of cancer diagnoses. If the annual study update form did not return, a repeated one or telephone would be conducted to contact the participant. Medical records were used as Supplementary material to certify the diagnoses. Family reports were collected if participants died, and death certificates, available autopsy reports, pathology slides, and pathology and other medical forms were used to ascertain the underlying cause of death (27), thus providing Supplementary material to certify the diagnoses.

### 2.5. Statistics analyses

For variables with <5% missing values, we used the modal value to impute the missing values for categorical covariates, including family history of any cancer, smoking status, aspirin consumption, ibuprofen consumption, history of diverticulitis, history of colorectal polyps, history of colon comorbidities, and family history of CRC. The median value was used to impute the continuous covariates, namely BMI and pack-years of smoking. For variables with more than 25% missing values, that is, physical activity level, we used the multiple imputation method to impute (Supplementary Table 2).

**Figure 2:**
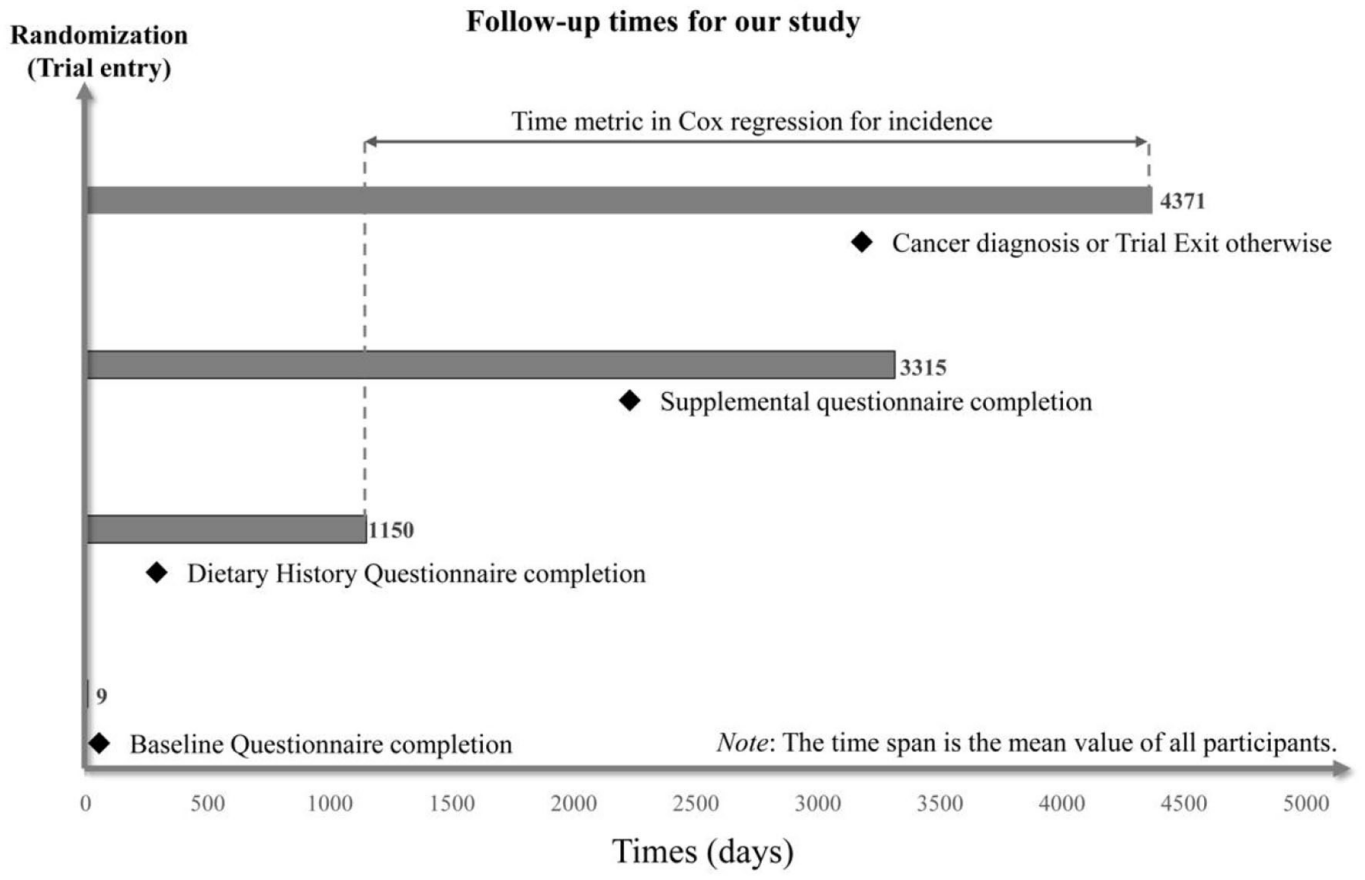
The timeline and follow-up scheme of our study.

Cox proportional hazards regression analyses were utilized to estimate hazard ratios (HRs) and 95% confidence intervals (CIs) for the associations between ELD adherence and subsite-specific CRC risk (colorectum, proximal colon, distal colon, and rectum). The follow-up period lasted from the completion of DHQ to the date of CRC diagnosis, death, loss to follow-up, and the end of follow-up (December 31, 2009), whichever came first (Figure 2) and was used as the time variable.

Based on ELD scores, we divided participants into quartiles and considered the first quartile to be the control group. The median scores of each quartile were assigned to each participant in the quartile to conduct Cox regression analyses and acquire P for trend. Some predefined confounders were included in the Cox regression models: Model 1 was adjusted for age, sex, and race. Model 2 was further adjusted for BMI, smoking status, pack-year of smoking, drinking status, physical activity level, regular consumption of aspirin, regular consumption of ibuprofen, family history of CRC, history of diverticulitis, history of colorectal polyps, history of colon comorbidities, energy intake from diet, protein intake from diet, carbohydrate intake from diet, and fat intake from diet. To further explore whether there was a linear dose-response association of ELD with CRC risk, we constructed a restricted cubic spline (RCS) model. Of note, we conducted the dose–response analysis in Model 2. Prespecified subgroup analyses were conducted to identify potential modifiers interacting with ELD, including age (≤65 vs. >65 years), sex (male vs. female), BMI (≤30 vs. >30 kg/m^2^), physical activity level (≤medium vs. >medium), smoking status (never vs. current/former), current alcohol drinking (yes vs. no), regular consumption of aspirin (yes vs. no), family history of colorectal cancer (yes vs. no), history of colorectal polyps (yes vs. no), history of colorectal comorbidities (yes vs. no), and energy intake from diet (≤medium vs. >medium). The following sensitivity analyses were conducted to testify to the robustness of our study: (1) First, we excluded participants with a history of diabetes, which is one of the risk factors for CRC (28); (2) Second, subjects with a history of heart attack were excluded; (3) third, the participants who had a colonoscopy or test for blood in stool in the past 3 years were excluded; (4) Finally, participants diagnosed in 2 years of follow-up were excluded.

## 3. Results

### 3.1. Baseline characteristics

A total of 98,415 participants aged 55–74 years were divided into quartiles according to their ELD scores [Q1 (ELD score ≤ 18), *n* = 25,468; Q2 (ELD score: 19–21), *n* = 26,969; Q3 (ELD score: 22-24), *n* = 25,611; Q4 (ELD score ≥ 25), *n* = 20,367]. As shown in Table 1, the mean age (SD) was 65.52 (5.73), and the mean ELD score (SD) was 21.19 (4.10). Higher ELD scores indicated greater adherence to the ELD. Among all included participants, we found that those who adhered more closely to ELD tended to be women (Q4, 62.05%; Q1, 38.69%) and non-white (Q4, 15.00%; Q1, 3.84%), have lower BMI (Q4, 26.21 ± 4.65 kg/m^2^; Q1, 27.84 ± 4.74 kg/m^2^), were more likely to be a non-smoker (never smoke: Q4, 52.95% vs. Q1, 41.60%), nondrinker (never drink: Q4, 30.83% vs. Q1, 25.13%), were less likely to use aspirin or ibuprofen regularly, and exercised more (Q4, 145.45 min/week vs. Q1, 104.30 min/week; Table 1). Consistent with expectation, mean daily intakes of emphasized components increased with higher adherence to ELD, while the opposite was observed for the intake of limited components. Furthermore, subjects in Q4 had a higher dietary intake of carbohydrates but a lower dietary intake of energy, protein, and total fat than in Q1 (Table 1).

**Table 1:** The baseline information of participants included in the study according to their EAT-Lancet diet scores^*^.

		**Quartiles of EAT-lancet diet scores**			
	**ALL**	**Q1 (** ≤ **18)**	**Q2 (19–21)**	**Q3 (22–24)**	**Q4 (**≥**25)**
	***N** =* **98,415**	***N** =* **25,468**	***N** =* **26,969**	***N** =* **25,611**	***N** =* **20,367**
**ELD score**	21.19 ± 4.10	16.07 ± 1.93	20.05 ± 0.81	22.92 ± 0.81	26.91 ± 2.02
**Age**	65.52 ± 5.73	64.75 ± 5.64	65.50 ± 5.74	65.88 ± 5.73	66.05 ± 5.73
**Sex**					
Male	47,183 (47.94%)	15,615 (61.31%)	13,219 (49.02%)	10,620 (41.47%)	7,729 (37.95%)
Female	51,232 (52.06%)	9,853 (38.69%)	13,750 (50.98%)	14,991 (58.53%)	12,638 (62.05%)
**Race**					
White	91,179 (92.65%)	24,491 (96.16%)	25,600 (94.92%)	23,777 (92.84%)	17,311 (85.00%)
Non-white	7,236 (7.35%)	977 (3.84%)	1,369 (5.08%)	1,834 (7.16%)	3,056 (15.00%)
**BMI (kg/m** ^**2**^ **)**	27.20 ± 4.79	27.84 ± 4.74	27.46 ± 4.79	27.07 ± 4.80	26.21 ± 4.65
**Smoke status**					
Never	47,216 (47.98%)	10,595 (41.60%)	12,851 (47.65%)	12,986 (50.70%)	10,784 (52.95%)
Current/former	51,199 (52.02%)	14,873 (58.40%)	14,118 (52.35%)	12,625 (49.30%)	9,583 (47.05%)
**Pack years of smoking**	17.49 ± 26.39	23.07 ± 30.44	17.68 ± 26.32	15.28 ± 24.29	13.03 ± 21.98
**Drinking**					
No	26,666 (27.10%)	6,399 (25.13%)	6,962 (25.81%)	7,026 (27.43%)	6,279 (30.83%)
Yes	71,749 (72.90%)	19,069 (74.87%)	20,007 (74.19%)	18,585 (72.57%)	14,088 (69.17%)
**Use aspirin regularly**					
No	52,218 (53.06%)	13,373 (52.51%)	14,323 (53.11%)	13,487 (52.66%)	11,035 (54.18%)
Yes	46,197 (46.94%)	12,095 (47.49%)	12,646 (46.89%)	12,124 (47.34%)	9,332 (45.82%)
**Use ibuprofen Regularly**					
No	70,843 (71.98%)	18,171 (71.35%)	19,209 (71.23%)	18,414 (71.90%)	15,049 (73.89%)
Yes	27,572 (28.02%)	7,297 (28.65%)	7,760 (28.77%)	7,197 (28.10%)	5,318 (26.11%)
**Physical activity (min/week)**	122.03 ± 108.98	104.30 ± 101.93	116.51 ± 105.53	126.85 ± 109.28	145.45 ± 116.69
**Arm**					
Intervention	50,113 (50.92%)	12,701 (49.87%)	13,715 (50.85%)	13,086 (51.10%)	10,611 (52.10%)
Control	48,302 (49.08%)	12,767 (50.13%)	13,254 (49.15%)	12,525 (48.90%)	9,756 (47.90%)
**Family history of colorectal cancer**					
No	86,008 (87.39%)	22,207 (87.20%)	23,481 (87.07%)	22,467 (87.72%)	17,853 (87.66%)
Yes/possibly	12,407 (12.61%)	3,261 (12.80%)	3,488 (12.93%)	3,144 (12.28%)	2,514 (12.34%)
**Had colonoscopy or test for blood in stool in past 3 years**					
No	55,017 (55.90%)	15,606 (61.28%)	15,089 (55.95%)	13,842 (54.05%)	10,480 (51.46%)
Yes	43,398 (44.10%)	9,862 (38.72%)	11,880 (44.05%)	11,769 (45.95%)	9,887 (48.54%)
**History of diverticulitis**					
No	91,783 (93.26%)	23,915 (93.90%)	25,082 (93.00%)	23,832 (93.05%)	18,954 (93.06%)
Yes	6,632 (6.74%)	1,553 (6.10%)	1,887 (7.00%)	1,779 (6.95%)	1,413 (6.94%)
**History of colon-related comorbidity**					
No	97,109 (98.67%)	25,113 (98.61%)	26,634 (98.76%)	25,272 (98.68%)	20,090 (98.64%)
Yes	1,306 (1.33%)	355 (1.39%)	335 (1.24%)	339 (1.32%)	277 (1.36%)
**History of colorectal polyps**					
No	91,874 (93.35%)	23,791 (93.42%)	25,166 (93.31%)	23,926 (93.42%)	18,991 (93.24%)
Yes	6,541 (6.65%)	1,677 (6.58%)	1,803 (6.69%)	1,685 (6.58%)	1,376 (6.76%)
**History of hypertension**					
No	66,613 (67.69%)	17,388 (68.27%)	18,078 (67.03%)	17,181 (67.08%)	13,966 (68.57%)
Yes	31,802 (32.31%)	8,080 (31.73%)	8,891 (32.97%)	8,430 (32.92%)	6,401 (31.43%)
**History of heart attack**					
No	90,356 (91.81%)	23,332 (91.61%)	24,820 (92.03%)	23,537 (91.90%)	18,667 (91.65%)
Yes	8,059 (8.19%)	2,136 (8.39%)	2,149 (7.97%)	2,074 (8.10%)	1,700 (8.35%)
Food energy from diet (kcal/day)	1728.59 ± 658.00	1874.06 ± 694.00	1749.03 ± 660.54	1652.40 ± 625.11	1615.41 ± 611.27
Protein from diet (g/day)	96.48 ± 18.29	97.25 ± 19.08	96.84 ± 18.39	96.60 ± 17.86	94.90 ± 17.56
Carbohydrate from diet (g/day)	324.96 ± 58.52	302.29 ± 53.43	318.98 ± 55.64	332.99 ± 55.97	351.11 ± 59.04
Total fat from diet (g/day)	88.28 ± 20.88	95.44 ± 19.19	89.67 ± 19.95	85.58 ± 20.44	80.88 ± 21.53
Fiber intake from diet (g/day)	11.86 ± 5.45	9.46 ± 4.06	11.24 ± 4.69	12.54 ± 5.28	14.82 ± 6.44
**Components of ELD score (g/day)**					
Vegetables	315.39 ± 221.43	183.93 ± 123.41	274.36 ± 167.06	361.36 ± 213.12	476.29 ± 267.26
Fruits	276.34 ± 218.69	149.09 ± 135.33	247.30 ± 183.02	322.80 ± 210.57	415.46 ± 254.27
Unsaturated oils	2.39 ± 4.72	1.79 ± 3.34	2.02 ± 3.75	2.36 ± 4.50	3.64 ± 6.88
Legumes	52.92 ± 61.84	27.64 ± 25.81	42.57 ± 37.82	58.36 ± 53.21	91.40 ± 98.65
Nuts	13.45 ± 19.04	8.60 ± 11.61	11.48 ± 15.78	14.18 ± 19.50	21.21 ± 26.09
Whole grains	94.15 ± 91.02	55.37 ± 51.28	79.95 ± 71.12	103.95 ± 89.74	149.13 ± 120.96
Fish	22.84 ± 24.71	14.74 ± 15.93	20.09 ± 19.77	25.18 ± 25.29	33.66 ± 33.08
Beef and lamb	53.58 ± 33.15	65.23 ± 35.35	57.56 ± 32.23	50.56 ± 29.95	37.53 ± 28.01
Pork	8.59 ± 10.88	12.26 ± 13.63	9.35 ± 10.75	7.22 ± 8.93	4.72 ± 7.17
Poultry	52.45 ± 53.91	51.76 ± 51.48	54.08 ± 53.67	53.96 ± 55.19	49.27 ± 55.39
Eggs	20.98 ± 25.39	29.71 ± 30.09	22.04 ± 25.40	17.77 ± 22.36	12.68 ± 17.91
Dairy	399.51 ± 373.77	471.35 ± 423.48	421.29 ± 382.57	381.42 ± 348.62	303.59 ± 295.80
Potatoes	87.49 ± 65.32	106.49 ± 73.75	92.51 ± 65.60	81.36 ± 59.05	64.81 ± 51.87
Added sugar	71.16 ± 37.21	84.34 ± 45.15	73.33 ± 36.51	66.58 ± 31.42	57.59 ± 26.50

* Values are mean ± standard deviation or counts (percentage) as indicated.

### 3.2. ELD scores and CRC incidence

During a mean follow-up of 8.82 years, we documented 1054 CRC cases, which consisted of 626 proximal colon cancers, 214 distal colon cancers, and 194 rectal cancers. Compared with those in the lowest quartile (Q1), participants in the highest quartile of ELD scores (Q4) had a decreased CRC risk after adjusting for potential CRC risk factors (HR_Q4vs.Q1_: 0.81; 95% CI: 0.67, 0.98; P-trend = 0.034; Table 2). We did not record any significant association between the ELD scores and anatomic CRC (proximal colon cancer: HR_Q4vs.Q1_: 0.85; 95% CI: 0.67, 1.09; P-trend = 0.160; distal colon cancer: HR_Q4vs.Q1_: 0.73; 95% CI: 0.47, 1.12; P-trend = 0.258; rectal cancer: 0.69; 95% CI: 0.43, 1.11; P-trend = 0.205; Supplementary Table 3).

**Table 2:** Hazard ratios of the association of the EAT-Lancet diet score with the risk of colorectal cancer.

**Quartiles of ELD scores**	**No. of participants**	**No. of cases**	**Person-years**	**Hazard ratio (95% confidence interval)**		
				**Unadjusted**	**Model 1** ^a^	**Model 2** ^b^
Quartile 1 (≤18)	25468	314	221736.80	1.00 (reference)	1.00 (reference)	1.00 (reference)
Quartile 2 (19–21)	26969	273	238353.80	0.81 (0.69, 0.95)	0.79 (0.67, 0.93)	0.81 (0.69, 0.95)
Quartile 3 (22–24)	25611	266	227230.70	0.83 (0.70, 0.97)	0.80 (0.68, 0.94)	0.84 (0.70, 0.99)
Quartile 4 (≥25)	20367	201	180807.70	0.79 (0.66, 0.94)	0.74 (0.62, 0.89)	0.81 (0.67, 0.98)
P for trend				0.009	0.001	0.034

a Adjusted for age (years), sex (male, female), and race (white, non-white).

b Adjusted for model 1 plus body mass index (kg/m^2^), smoking status (never, current or former), pack-year of smoking, drinking status (no, yes), physical activity level (min/week), aspirin and ibuprofen consumption (no, yes), family history of colorectal cancer (no, yes), history of diverticulitis (no, yes), colorectal polyps (no, yes), colon comorbidities (including ulcerative colitis, Crohn's disease, Gardner's syndrome, or familial polyposis) (no, yes), energy intake from diet (kcal/day), protein intake from diet (g/day), carbohydrate intake from diet (g/day), and fat intake from diet (g/day).

### 3.3. Additional analyses

**Figure 3:**
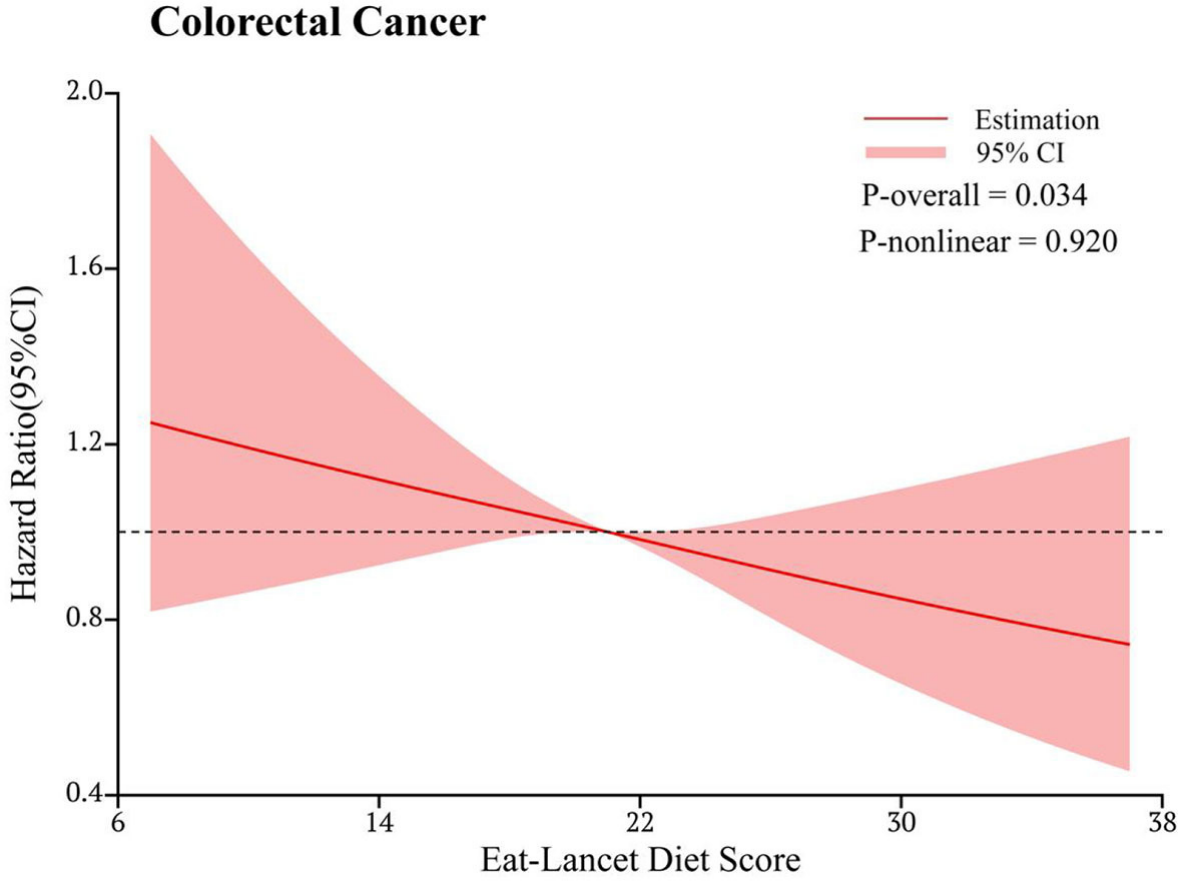
Dose-response analysis of the association of the EAT-Lancet diet score with the risk of colorectal cancer. The hazard ratio was adjusted for age (years), sex (male and female), race (white and non-white), body mass index (kg/m^2^), smoking status (never, current or former), pack-year of smoking, drinking status (no, yes), physical activity level (min/week), aspirin and ibuprofen consumption (no, yes), family history of colorectal cancer (no, yes), history of diverticulitis (no, yes), colorectal polyps (no, yes), colon comorbidities (including ulcerative colitis, Crohn's disease, Gardner's syndrome, or familial polyposis) (no, yes), energy intake from the diet (kcal/day), protein intake from the diet (g/day), carbohydrate intake from the diet (g/day), and fat intake from the diet (g/day).

In the RCS model, we found a linear association between the ELD score and CRC incidence (P-nonlinearity = 0.920) (Figure 3). Subgroup analyses showed no significant modifiers interacting with ELD, including age, sex, BMI, physical activity level, smoking status, drinking status, regular consumption of aspirin, family history of CRC, history of colorectal polyps, history of colorectal comorbidities, and energy intake from diet (all P-interaction > 0.05; Table 3).

**Table 3:** Subgroup analyses on the association of the EAT-Lancet diet scores with the risk of colorectal cancer.

**Subgroup variable**	**No. of cases**		**Hazard ratio (95% confidence interval) EAT-lancet diet scores**				**P for trend**	**P for interaction**
		**Person-years**	**Quartile 1 (** ≤ **18)**	**Quartile 2 (19–21)**	**Quartile 3 (22–24)**	**Quartile 4 (**≥**25)**		
**Age (years)**								0.528
≤65	384	454117.86	1.00 (reference)	0.70 (0.54, 0.92)	0.82 (0.63, 1.09)	0.74 (0.54, 1.02)	0.068	
>65	670	414011.15	1.00 (reference)	0.89 (0.72, 1.10)	0.87 (0.70, 1.08)	0.87 (0.68, 1.10)	0.214	
**Sex**								0.270
Male	571	411493.40	1.00 (reference)	0.88 (0.72, 1.09)	0.88 (0.70, 1.10)	0.74 (0.56, 0.97)	0.037	
Female	483	456635.61	1.00 (reference)	0.70 (0.54, 0.92)	0.77 (0.60, 1.00)	0.83 (0.63, 1.09)	0.344	
**Body mass index (kg/m** ^**2**^ **)**								0.582
≤30	799	675555.72	1.00 (reference)	0.80 (0.66, 0.97)	0.84 (0.69, 1.03)	0.76 (0.61, 0.95)	0.027	
>30	255	192573.28	1.00 (reference)	0.84 (0.61, 1.15)	0.80 (0.57, 1.13)	0.96 (0.65, 1.42)	0.523	
**Physical activity (min/week)**								0.299
≤medium^a^	651	430488.15	1.00 (reference)	0.84 (0.69, 1.03)	0.77 (0.62, 0.96)	0.82 (0.64, 1.06)	0.035	
>medium^a^	403	437640.85	1.00 (reference)	0.77 (0.57, 1.03)	0.95 (0.72, 1.26)	0.81 (0.59, 1.10)	0.439	
**Smoking status**								0.876
Never	470	422690.59	1.00 (reference)	0.75 (0.59, 0.97)	0.82 (0.64, 1.06)	0.79 (0.60, 1.05)	0.182	
Current/Former	584	445438.42	1.00 (reference)	0.85 (0.69, 1.06)	0.84 (0.67, 1.06)	0.81 (0.62, 1.05)	0.082	
**Drinker**								0.425
No	279	233353.73	1.00 (reference)	0.70 (0.50, 0.99)	0.87 (0.62, 1.23)	0.87 (0.58, 1.30)	0.744	
Yes	775	634775.27	1.00 (reference)	0.86 (0.71, 1.03)	0.83 (0.68, 1.02)	0.81 (0.64, 1.01)	0.052	
**Regular consumption of aspirin**								0.524
No	578	464200.11	1.00 (reference)	0.76 (0.60, 0.95)	0.85 (0.68, 1.07)	0.78 (0.60, 1.01)	0.119	
Yes	476	403928.90	1.00 (reference)	0.87 (0.68, 1.10)	0.81 (0.62, 1.04)	0.83 (0.62, 1.10)	0.124	
**Family history of colorectal cancer**								0.611
No	895	758790.68	1.00 (reference)	0.83 (0.69, 0.99)	0.82 (0.68, 0.98)	0.81(0.66, 1.00)	0.044	
Yes/possible	159	109338.33	1.00 (reference)	0.69 (0.45, 1.06)	0.92 (0.61, 1.41)	0.74 (0.45, 1.22)	0.440	
**History of colorectal polyps**								0.694
No	963	810317.57	1.00 (reference)	0.81 (0.68, 0.96)	0.83 (0.69, 0.99)	0.79 (0.65, 0.97)	0.026	
Yes	91	57811.44	1.00 (reference)	0.78 (0.43, 1.41)	0.91 (0.51, 1.63)	1.00 (0.53, 1.88)	0.893	
**History of colorectal comorbidities**								0.455
No	1038	856685.97	1.00 (reference)	0.80 (0.68, 0.95)	0.82 (0.69, 0.98)	0.81 (0.67, 0.98)	0.033	
Yes	16	11443.03	1.00 (reference)	1.84 (0.41, 8.17)	2.20 (0.50, 9.71)	0.71 (0.10, 5.00)	0.991	
**Energy intake from the diet (kcal/day)**								0.517
≤Medium^b^	535	434541.58	1.00 (reference)	0.82 (0.64, 1.04)	0.92 (0.72, 1.17)	0.84 (0.64, 1.09)	0.379	
>Medium^b^	519	433587.42	1.00 (reference)	0.82 (0.65, 1.02)	0.75 (0.59, 0.96)	0.79 (0.60, 1.04)	0.029	

The hazard ratio was adjusted for age (years), sex (male, female), race (white, non-white), body mass index (kg/m^2^), smoking status (never, current or former), pack-year of smoking, drinking status (no, yes), physical activity level (min/week), aspirin and ibuprofen consumption (no, yes), family history of colorectal cancer (no, yes), history of diverticulitis (no, yes), colorectal polyps (no, yes), colon comorbidities (including ulcerative colitis, Crohn's disease, Gardner's syndrome, or familial polyposis) (no, yes), energy intake from diet (kcal/day), protein intake from diet (g/day), carbohydrate intake from diet (g/day), and fat intake from diet (g/day).

a The median of physical activity is 104 min/week.

b The median dietary energy intake in this study is 1615 kcal/day.

After excluding participants with a history of diabetes, a history of heart attack, those who had colonoscopy or test for blood in stool in the past 3 years, and those diagnosed with CRC in 2 years, the inverse association between the ELD score and CRC incidence still existed (all P-trend < 0.05), which demonstrated the robustness of our finding (Table 4).

**Table 4:** Sensitivity analyses on the association of the EAT-Lancet diet scores with the risk of overall colorectal cancer^a^.

**Categories**	**No. of participants**	**No. of cases**	**Hazard ratio (95% confidence interval) of the EAT-lancet diet Scores** ^a^				
			**Quartile 1 (** ≤ **18)**	**Quartile 2 (19–21)**	**Quartile 3 (22–24)**	**Quartile 4 (**≥**25)**	**P for trend**
Excluded participants with a history of diabetes^b^	91,950	952	1.00 (reference)	0.79 (0.67, 0.94)	0.82 (0.69, 0.98)	0.81 (0.66, 0.99)	0.044
Excluded participants with a history of heart attack^c^	90,356	953	1.00 (reference)	0.79 (0.66, 0.94)	0.83 (0.69, 0.99)	0.80 (0.65, 0.97)	0.035
Excluded participants had colonoscopy or test for blood in stool in the past 3 years^d^	55,017	594	1.00 (reference)	0.90 (0.73, 1.11)	0.83 (0.66, 1.04)	0.64 (0.48, 0.84)	0.001
Excluded participants diagnosed in 2 years	98,180	819	1.00 (reference)	0.84 (0.69, 1.01)	0.85 (0.70, 1.03)	0.79 (0.63, 0.98)	0.040
Repeated analysis of participants with unfilled data	98,415	1,054	1.00 (reference)	0.84 (0.69, 1.04)	0.79 (0.63, 0.98)	0.77 (0.60, 0.99)	0.024

a HR was adjusted for age, sex (male and female), race (white, non-white), body mass index (kg/m^2^), smoking status (never, current or former), pack-year of smoking, drinking status (no, yes), physical activity level (min/week), aspirin and ibuprofen consumption (no, yes), family history of colorectal cancer (no, yes), history of diverticulitis (no, yes), colorectal polyps (no, yes), colon comorbidities (no, yes), energy intake from diet (kcal/day), protein intake from diet (g/day), carbohydrate intake from diet (g/day), and fat intake from diet (g/day).

b The hazard ratio was not adjusted for a history of diabetes.

c The hazard ratio was not adjusted for a history of heart attack.

d Hazard ratio did not exclude participants who had colonoscopies or tests for blood in stool in the past 3 years.

## 4. Discussion

In the cohort of the PLCO Cancer Screening Trial, we used *a priori*-defined ELD score to assess adherence to ELD and evaluated its relationship with CRC risk. During a mean follow-up of 8.82 years, we found that greater adherence to the ELD was associated with a lower risk of CRC in a linear dose–response manner in American adults. Subgroup analyses showed no significant effect modifiers interacting with ELD on CRC, and our result was robust. We did not observe any significant association between ELD adherence and the risk of specific subsites of CRC.

In the realm of dietary approaches promoting health and sustainability, the ELD and the MD stand as two distinct paradigms marked by notable disparities in their historical origins and core principles. The ELD, guided by a global perspective and a resolute commitment to environmental sustainability, places a pronounced emphasis on the consumption of plant-based foods while advocating for a reduction in meat intake (7). In stark contrast, the MD remains region-specific, deeply entrenched in the rich culinary traditions of Mediterranean nations, and underscores a well-balanced dietary pattern characterized by the incorporation of olive oil, whole grains, fruits, vegetables, and moderate portions of fish and poultry (28). The ELD's accentuation of plant-based foods, restricted consumption of meat and animal products, integration of sustainable agricultural practices, and adaptability to diverse cultural contexts collectively position it as a dietary choice with enhanced environmental sustainability. While both diets share commonalities in their promotion of healthful and sustainable eating habits, the fundamental principles of the ELD take precedence in prioritizing the reduction of the environmental impact associated with dietary choices. Thus, the ELD emerges as an appealing option for individuals seeking a dietary approach that promotes health and demonstrates a steadfast commitment to sustainable practices on a global scale.

With improved living standards, residents' income, and urbanization, eating behavior of humans is gradually shifting to unhealthy diets that are high-energy, high-animal-origin, and ultra-processed (7). This dietary habit is threatening human health and environmental sustainability, and an unhealthy diet has become the largest burden to diseases and premature death, surpassing smoking and drinking (7). Therefore, in 2019, the EAT–Lancet Commission proposed a plant-based diet that was good for human health and environmental sustainability (7). The effectiveness of ELD has been confirmed by many studies. In terms of environmental sustainability, a meta-analysis by Springmann et al. (14) showed that compliance with the ELD was associated with a 42% reduction in greenhouse gas emissions and a 10% reduction in freshwater consumption. These advantages were further confirmed by the study of Cambeses-Franco et al. (29). In terms of human health, ELD has been believed to decrease the incidence and mortality from non-communicable diseases (NCDs) (7, 16, 18, 24, 29–31). In the Malmö Diet and Cancer study cohort, adherence to ELD can reduce the risk of type 2 diabetes by 18% (30). In the Swedish population, adherence to ELD can reduce the risk of coronary events by 20% (31) and can reduce the risk of all-cause mortality by 25%, cardiovascular disease death by 32%, and cancer-related death by 24% (24).

In previous research on ELD, only two studies involved the incidence of cancer (18, 32). Research by Laine et al. (32) showed that adherence to ELD over a 20-year period could effectively decrease 10%-39% of cancer risk in a large prospective cohort of the European Prospective Investigation into Cancer and Nutrition (EPIC). A prospective cohort study of the French population focused for the first time on ELD adherence and the risk of specific types of cancer. They found that adherence to ELD was associated with a decreased risk of lung cancer while not associated with the risk of breast cancer, prostate cancer, and CRC (18). In our study, we found a statistically significant association between adherence to ELD and the risk of CRC. The possible reason may be that the study populations were different: the study of Berthy et al. (18) was conducted in France, while our study was conducted in America. There was a difference in their dietary habits. Compared to the French, Americans are less likely to eat fruits and vegetables, which are determined by their respective cultural background (33).

ELD emphasizes the intake of vegetables, whole grains, fruits, unsaturated oils, legumes, nuts, and fish and limits the intake of beef and lamb, pork, poultry, eggs, potatoes, dairy, and added sugar. All the emphasized food components have been proven to reduce the CRC risk (34–38), and most of the restricted components, such as beef and lamb, pork, eggs, potatoes, and added sugar (6, 39–43), were reported to increase the CRC incidence. As for dairy, there are many types. Certain types, such as cheese and low-fat dairy, contribute to the prevention of CRC, while whole-fat dairy, which is the component we used to construct the ELD score, may increase the risk of CRC (44). The impact of an individual diet on disease is limited, but the synergies and interactions between multiple diets, combined with long-term accumulation, may eventually contribute to the onset, delay, or prevention of NCDs (45–47).

The occurrence of CRC is a heterogeneous process that is influenced by the environment, microbial exposure, diet, and host immunity. Evidence suggests that CRC is caused by gradual interference with changes in gut microbiota composition attributed to food composition or diet and changes in oncogenes and tumor suppressor genes (48). Intestinal microorganisms can promote CRC development by metabolizing food to produce different substances and causing chronic inflammation, affecting host immunity and genetic susceptibility of the body (48). These may help explain the impact of food on CRC.

Our study has some limitations. First, our study had fully adjusted covariates available in the PLCO Cancer Screening Trial. However, we could not rule out the possibility that our finding was biased by unmeasured or unrecognized confounders. Second, all diet-associated information used to calculate the ELD score was assessed using a questionnaire that was only collected once at baseline, without considering the change of dietary habits over time. However, a study has suggested that compared to assessing a dietary pattern using the cumulative averages, baseline diet data can help acquire a similar statistical association for disease risk analysis (49). Third, we found no significant interaction in the incidence of CRC between the ELD score and potential effect modifiers in subgroup analyses, so we cannot provide guidance for specific subgroups based on our results. Fourth, in the Cox regression analyses of subsite CRC, there was no statistically significant association between the ELD score and proximal colon cancer, distal colon cancer, or distal cancer. The reason may be attributed to the limited number of cancer cases in the proximal colon, distal colon, and rectum, leading to insufficient statistical power for these analyses. Finally, this study was conducted on Americans aged 55–74 years. It is unknown whether the result can be extended to populations of other ages or countries with different physical characteristics, dietary cultures, and genetic backgrounds, so more studies need to be conducted.

## 5. Conclusion

In conclusion, in American adults, great adherence to ELD is associated with decreased CRC risk in a linear dose-response manner. Our result supports the role of ELD in preventing CRC, which provides new evidence for ELD in cancer prevention. Therefore, it is crucial to publicize the ELD.

## Data availability statement

The data analyzed in this study is subject to the following licenses/restrictions: The raw data used in this article is not available because of the National Cancer Institute's data policy. Requests to access these datasets should be directed to NCIinfo@nih.gov.

## Ethics statement

The studies involving humans were approved by the NCI (Project ID: PLCO-1231). The studies were conducted in accordance with the local legislation and institutional requirements. Written informed consent for participation was not required from the participants or the participants' legal guardians/next of kin in accordance with the national legislation and institutional requirements.

## Author contributions

XR: Writing—review and editing, Data curation, Investigation, Writing—original draft. CY: Data curation, Writing—original draft. LP: Funding acquisition, Writing—review and editing, Project administration. HG: Funding acquisition, Writing—review and editing. YX: Writing—review and editing. YT: Writing—review and editing. HH: Writing—review and editing. LX: Writing—review and editing. YW: Writing—review and editing, Funding acquisition. YJ: Writing—review and editing.

## References

[1] Siegel RL Wagle NS Cercek A Smith RA Jemal A. Colorectal cancer statistics, 2023. CA Cancer J Clin. (2023) 73:233–54. 10.3322/caac.21772 36856579

[2] Kuipers EJ Grady WM Lieberman D Seufferlein T Sung JJ Boelens PG. Colorectal cancer. Nat Rev Dis Primer. (2015) 1:15065. 10.1038/nrdp.2015.65 PMC4874655 27189416

[3] Bradbury KE Murphy N Key TJ. Diet and colorectal cancer in UK Biobank: a prospective study. Int J Epidemiol. (2020) 49:246–58. 10.1093/ije/dyz064 30993317 PMC7124508

[4] Schwingshackl L Schwedhelm C Hoffmann G Knüppel S Laure Preterre A Iqbal K. Food groups and risk of colorectal cancer. Int J Cancer. (2018) 142:1748–58. 10.1002/ijc.31198 29210053

[5] Vieira AR Abar L Chan DSM Vingeliene S Polemiti E Stevens C. Foods and beverages and colorectal cancer risk: a systematic review and meta-analysis of cohort studies, an update of the evidence of the WCRF-AICR Continuous Update Project. Ann Oncol Off J Eur Soc Med Oncol. (2017) 28:1788–802. 10.1093/annonc/mdx171 28407090

[6] Yuan C Joh H-K Wang Q-L Zhang Y Smith-Warner SA Wang M. Sugar-sweetened beverage and sugar consumption and colorectal cancer incidence and mortality according to anatomic subsite. Am J Clin Nutr. (2022) 115:1481–9. 10.1093/ajcn/nqac040 35470384 PMC9170474

[7] Willett W Rockström J Loken B Springmann M Lang T Vermeulen S. Food in the Anthropocene: the EAT-Lancet Commission on healthy diets from sustainable food systems. Lancet Lond Engl. (2019) 393:447–92. 10.1016/S0140-6736(18)31788-4 30660336

[8] Hirvonen K Bai Y Headey D Masters WA. Affordability of the EAT-Lancet reference diet: a global analysis. Lancet Glob Health. (2020) 8:e59–66. 10.1016/S2214-109X(19)30447-4 31708415 PMC7024996

[9] Schulpen M van den Brandt PA. Mediterranean diet adherence and risk of colorectal cancer: the prospective Netherlands. Cohort Study Eur J Epidemiol. (2020) 35:25. 10.1007/s10654-019-00549-8 31494792 PMC7058569

[10] Cancer Mediterranean Diet: A Review - PubMed. (2023). Available online at: https://pubmed.ncbi.nlm.nih.gov/31480794/ (accessed July 6, 2023).

[11] Trichopoulou A Costacou T Bamia C Trichopoulos D. Adherence to a Mediterranean diet and survival in a Greek population. N Engl J Med. (2003) 348:2599–608. 10.1056/NEJMoa025039 12826634

[12] Barrea L Pugliese G Laudisio D Colao A Savastano S Muscogiuri G. Mediterranean diet as medical prescription in menopausal women with obesity: a practical guide for nutritionists. Crit Rev Food Sci Nutr. (2021) 61:1201–11. 10.1080/10408398.2020.1755220 32329636

[13] Vanham D Guenther S Ros-Baró M Bach-Faig A. Which diet has the lower water footprint in Mediterranean countries? Resour Conserv Recycl. (2021) 171:105631. 10.1016/j.resconrec.2021.105631 34345116 PMC8216694

[14] Springmann M Spajic L Clark MA Poore J Herforth A Webb P. The healthiness and sustainability of national and global food based dietary guidelines: modelling study. BMJ. (2020) 370:m2322. 10.1136/bmj.m2322 32669369 PMC7362232

[15] Lazarova SV Sutherland JM Jessri M. Adherence to emerging plant-based dietary patterns and its association with cardiovascular disease risk in a nationally representative sample of Canadian adults. Am J Clin Nutr. (2022) 116:57–73. 10.1093/ajcn/nqac062 35265975 PMC9257478

[16] Ibsen DB Christiansen AH Olsen A Tjønneland A Overvad K Wolk A. Adherence to the EAT-lancet diet and risk of stroke and stroke subtypes: a cohort study. Stroke. (2022) 53:154–63. 10.1161/STROKEAHA.121.036738 34872335

[17] Cacau LT Benseñor IM Goulart AC Cardoso L de O Santos I de S Lotufo PA. Adherence to the EAT-Lancet sustainable reference diet and cardiometabolic risk profile: cross-sectional results from the ELSA-Brasil cohort study. Eur J Nutr. (2023) 62:807–17. 10.1007/s00394-022-03032-5 36266476

[18] Berthy F Brunin J Allès B Fezeu LK Touvier M Hercberg S. Association between adherence to the EAT-Lancet diet and risk of cancer and cardiovascular outcomes in the prospective NutriNet-Santé cohort. Am J Clin Nutr. (2022) 116:980–91. 10.1093/ajcn/nqac208 35918246

[19] Gohagan JK Prorok PC Greenwald P Kramer BS. The PLCO cancer screening trial: background, goals, organization, operations, results. Rev Recent Clin Trials. (2015) 10:173–80. 10.2174/1574887110666150730123004 26238115

[20] Zhu CS Pinsky PF Kramer BS Prorok PC Purdue MP Berg CD. The prostate, lung, colorectal, and ovarian cancer screening trial and its associated research resource. J Natl Cancer Inst. (2013) 105:1684–93. 10.1093/jnci/djt281 24115361 PMC3888207

[21] Miller AB Feld R Fontana R Gohagan JK Jatoi I Lawrence W. Changes in and impact of the death review process in the prostate, lung, colorectal and ovarian (PLCO) cancer screening trial. Rev Recent Clin Trials. (2015) 10:206–11. 10.2174/1574887110666150730120752 26238119

[22] Shan Z Guo Y Hu FB Liu L Qi Q. Association of low-carbohydrate and low-fat diets with mortality among US adults. JAMA Intern Med. (2020) 180:513–23. 10.1001/jamainternmed.2019.6980 31961383 PMC6990856

[23] Csizmadi I Boucher BA Lo Siou G Massarelli I Rondeau I Garriguet D. Using national dietary intake data to evaluate and adapt the US Diet History Questionnaire: the stepwise tailoring of an FFQ for Canadian use. Public Health Nutr. (2016) 19:3247–55. 10.1017/S1368980016001506 27349130 PMC10271078

[24] Stubbendorff A Sonestedt E Ramne S Drake I Hallström E Ericson U. Development of an EAT-Lancet index and its relation to mortality in a Swedish population. Am J Clin Nutr. (2022) 115:705–16. 10.1093/ajcn/nqab369 34791011 PMC8895215

[25] Botteri E Peveri G Berstad P Bagnardi V Chen SLF Sandanger TM. Changes in lifestyle and risk of colorectal cancer in the european prospective investigation into cancer and nutrition. Am J Gastroenterol. (2023) 118:702–11. 10.14309/ajg.0000000000002065 36227801

[26] Yu Y-C Paragomi P Jin A Wang R Schoen RE Koh W-P. Low-carbohydrate diet score and the risk of colorectal cancer: findings from the Singapore Chinese health study. Cancer Epidemiol Biomark Prev Publ Am Assoc Cancer Res Cosponsored Am Soc Prev Oncol. (2023) 32:802–8. 10.1158/1055-9965.c.6573874.v1 36944231 PMC10239354

[27] Prorok PC Andriole GL Bresalier RS Buys SS Chia D Crawford ED. Design of the prostate, lung, colorectal and ovarian (PLCO) cancer screening trial. Control Clin Trials. (2000) 21:273S–309S. 10.1016/S0197-2456(00)00098-2 11189684

[28] Colorectal Colorectal Cancer: A Review of Carcinogenesis Global Epidemiology Current Challenges Risk Factors Preventive Treatment Strategies - PubMed. (2023). Available online at: https://pubmed.ncbi.nlm.nih.gov/35406504/ (accessed September 10, 2023).

[29] Cambeses-Franco C Feijoo G Moreira MT González-García S. Co-benefits of the EAT-Lancet diet for environmental protection in the framework of the Spanish dietary pattern. Sci Total Environ. (2022) 836:155683. 10.1016/j.scitotenv.2022.155683 35526623

[30] Zhang S Stubbendorff A Olsson K Ericson U Niu K Qi L. Adherence to the EAT-Lancet diet, genetic susceptibility, and risk of type 2 diabetes in Swedish adults. Metabolism. (2023) 141:155401. 10.1016/j.metabol.2023.155401 36682448

[31] Zhang S Dukuzimana J Stubbendorff A Ericson U Borné Y Sonestedt E. Adherence to the EAT-lancet diet and risk of coronary events in the Malmö diet and cancer cohort study. Am J Clin Nutr. (2023) 117:903–9. 10.1016/j.ajcnut.2023.02.018 36841443

[32] Laine JE Huybrechts I Gunter MJ Ferrari P Weiderpass E Tsilidis K. Co-benefits from sustainable dietary shifts for population and environmental health: an assessment from a large European cohort study. Lancet Planet Health. (2021) 5:e786–96. 10.1016/S2542-5196(21)00250-3 34688354 PMC8581185

[33] Kremer-Sadlik T Morgenstern A Peters C Beaupoil P Caët S Debras C. Eating fruits and vegetables An ethnographic study of American and French family dinners. Appetite. (2015) 89:84–92. 10.1016/j.appet.2015.01.012 25616214

[34] Aune D Chan DSM Lau R Vieira R Greenwood DC Kampman E. Dietary fibre, whole grains, and risk of colorectal cancer: systematic review and dose-response meta-analysis of prospective studies. BMJ. (2011) 343:d6617. 10.1136/bmj.d6617 22074852 PMC3213242

[35] Aune D Lau R Chan DSM Vieira R Greenwood DC Kampman E. Nonlinear reduction in risk for colorectal cancer by fruit and vegetable intake based on meta-analysis of prospective studies. Gastroenterology. (2011) 141:106–18. 10.1053/j.gastro.2011.04.013 21600207

[36] Watling CZ Schmidt JA Dunneram Y Tong TYN Kelly RK Knuppel A. Risk of cancer in regular and low meat-eaters, fish-eaters, and vegetarians: a prospective analysis of UK Biobank participants. BMC Med. (2022) 20:73. 10.1186/s12916-022-02256-w 35197066 PMC8867885

[37] Shen W Sun J Li Z Yao F Lin K Jiao X. Food intake and its effect on the species and abundance of intestinal flora in colorectal cancer and healthy individuals. Korean J Intern Med. (2021) 36:568–83. 10.3904/kjim.2019.373 33167104 PMC8137414

[38] Jin S Je Y. Nuts and legumes consumption and risk of colorectal cancer: a systematic review and meta-analysis. Eur J Epidemiol. (2022) 37:569–85. 10.1007/s10654-022-00881-6 35622305

[39] Carr PR Walter V Brenner H Hoffmeister M. Meat subtypes and their association with colorectal cancer: systematic review and meta-analysis. Int J Cancer. (2016) 138:293–302. 10.1002/ijc.29423 25583132

[40] Song M Garrett WS Chan AT. Nutrients, foods, and colorectal cancer prevention. Gastroenterology. (2015) 148:1244–60. 10.1053/j.gastro.2014.12.035 25575572 PMC4409470

[41] Mejborn H Møller SP Thygesen LC Biltoft-Jensen A. Dietary intake of red meat, processed meat, and poultry and risk of colorectal cancer and all-cause mortality in the context of dietary guideline compliance. Nutrients. (2020) 13:32. 10.3390/nu13010032 33374887 PMC7823645

[42] Åsli LA Olsen A Braaten T Lund E Skeie G. Potato consumption and risk of colorectal cancer in the Norwegian women and cancer cohort. Nutr Cancer. (2017) 69:564–72. 10.1080/01635581.2017.1295086 28323437

[43] Shen J Li Y Xu M Wu F Jiang Y Liu X. Association of egg consumption with colorectal polyp prevalence: findings from the Lanxi Pre-Colorectal Cancer Cohort (LP3C) in China. Food Funct. (2023) 14:2597–606. 10.1039/D2FO03061F 36847183

[44] Gil H Chen Q-Y Khil J Park J Na G Lee D. Milk intake in early life and later cancer risk: a meta-analysis. Nutrients. (2022) 14:1233. 10.3390/nu14061233 35334890 PMC8948718

[45] Fung TT Chiuve SE McCullough ML Rexrode KM Logroscino G Hu FB. Adherence to a DASH-style diet and risk of coronary heart disease and stroke in women. Arch Intern Med. (2008) 168:713–20. 10.1001/archinte.168.7.713 18413553

[46] Milenkovic D Declerck K Guttman Y Kerem Z Claude S Weseler AR. (-)-Epicatechin metabolites promote vascular health through epigenetic reprogramming of endothelial-immune cell signaling and reversing systemic low-grade inflammation. Biochem Pharmacol. (2020) 173:113699. 10.1016/j.bcp.2019.113699 31756325

[47] Peanut Consumption Improves Indices of Cardiovascular Disease Risk in Healthy Adults - PubMed. (2023). Available online at: https://pubmed.ncbi.nlm.nih.gov/12672709/ (accessed May 8, 2023). 10.1080/07315724.2003.10719286 12672709

[48] O'Keefe SJD. Diet, microorganisms and their metabolites, and colon cancer. Nat Rev Gastroenterol Hepatol. (2016) 13:691–706. 10.1038/nrgastro.2016.165 27848961 PMC6312102

[49] Hu FB Stampfer MJ Rimm E Ascherio A Rosner BA Spiegelman D. Dietary fat and coronary heart disease: a comparison of approaches for adjusting for total energy intake and modeling repeated dietary measurements. Am J Epidemiol. (1999) 149:531–40. 10.1093/oxfordjournals.aje.a009849 10084242

