# Supplementary material for: Compliance with the EAT-Lancet diet and risk of colorectal cancer: a prospective cohort study in 98,415 American adults
Source: Front Nutr. 2023 Oct 19;10:1264178. doi: 10.3389/fnut.2023.1264178 (PMC10621045; doi:10.3389/fnut.2023.1264178)
Supplement: Supplementary file 1 [file Data_Sheet_1.PDF]

Supplementary Material

The compliance to EAT-Lancet diet and risk of colorectal cancer: a prospective cohort study in 98,415 American adults

Xiaorui Ren [1†], Chuanchuan Yu [2†], Linglong Peng [1], Haitao Gu[1], Yi Xiao [1], Yunhao Tang [1], Hongmei He [1], Ling Xiang [3*], Yaxu Wang [1*], Yahui Jiang [1*]

Supplementary Table 1. Criteria for determining the EAT-Lancet diet scores

**Food components of EAT-Lancet diet[1]**		**Target intake (reference interval)[2]**	**3 points**	**2 points**	**1 point**	**0 points**
**Emphasized intake**	Vegetables	300 (200–600)	>300	200–300	100–200	<100
	Fruits	200 (100–300)	>200	100–200	50–100	<50
	Unsaturated oils	40 (20–80)	>40	20–40	10–20	<10
	Legumes	75 (0–150)	>75	37.5–75	18.75–37.5	<18.75
	Nuts	50 (0–100)	>50	25–50	12.5–25	<12.5
	Whole grains	232	>232	116–232	58–116	<58
	Fish	28 (0–100)	>28	14–28	7–14	<7
**Limited intake**	Beef and lamb	7 (0–14)	<7	7–14	14–28	>28
	Pork	7 (0–14)	<7	7–14	14–28	>28
	Poultry	29 (0–58)	<29	29–58	58–116	>116
	Eggs	13 (0–25)	<13	13–25	25–50	>50
	Dairy	250 (0–500)	<250	250–500	500–1000	>1000
	Potatoes	50 (0–100)	<50	50–100	100–200	>200
	Added sugar	31 (0–31)	<31	31–62	62–124	>124

1 Food components in the index are based on the EAT-Lancet diet as grams per day.

2 Target and reference values from the EAT-Lancet diet, based on an energy intake of 2500 kcal, expressed in grams (1).

Supplementary Table 2. Distribution of variables with missing data before and after imputation

Variable	Before imputation	After imputation	Number (%) with missing data
**Family history of colorectal cancer**			757 (0.77%)
No	85251 (87.30%)	86008 (87.39%)	
Yes	10019 (10.26%)	10019 (10.18%)	
Possibly	2388 (2.44%)	2388 (2.43%)	
**Body mass index (kg/m^2)**	27.21±4.82	27.20±4.79	1293 (1.31%)
**Smoking status**			20 (0.02%)
Never	47196 (47.97%)	47216 (47.98%)	
Current	8987 (9.13%)	8987 (9.13%)	
Former	42212 (42.90%)	42212 (42.89%)	
**Smoking pack-years**	17.66±26.49	17.49±26.39	1104 (1.12%)
**Aspirin consumption**			426 (0.43%)
No	51792 (52.85%)	52218 (53.06%)	
Yes	46197 (47.15%)	46197 (46.94%)	
**Ibuprofen consumption**			422 (0.43%)
No	70421 (71.86%)	70843 (71.98%)	
Yes	27572 (28.14%)	27572 (28.02%)	
**History of diverticulitis**			623 (0.63%)
No	91160 (93.22%)	91783 (93.26%)	
Yes	6632 (6.78%)	6632 (6.74%)	
**History of colon comorbidities**			863 (0.88%)
No	96246 (98.66%)	97109 (98.67%)	
Yes	1306 (1.34%)	1306 (1.33%)	
**History of colorectal polyps**			602 (0.61%)
No	91272 (93.31%)	91874 (93.35%)	
Yes	6541 (6.69%)	6541 (6.65%)	
**Had colonoscopy or test for blood in stool in past 3 years**			3137 (3.19%)
No	51880 (54.45%)	55017 (55.90%)	
Yes	43398 (45.55%)	43398 (44.10%)	
**Physical activity (min/week)**	125.16±123.33	122.08±109.00	24900 (25.30%)

Supplementary Table 3. EAT-Lancet diet and the risk of colorectal cancer by anatomical site

variable	No. of cases	EAT-Lancet diet score				P for trend
		Q1 (≤18)	Q2 (19-21)	Q3 (22-24)	Q4 (≥25)	
**Proximal colon cancer**	626	173	169	156	128	
Unadjusted		1.00 (reference)	0.91 (0.74, 1.12)	0.88 (0.71, 1.09)	0.91 (0.72, 1.14)	0.341
Model 1[a]		1.00 (reference)	0.86 (0.69, 1.06)	0.81 (0.65, 1.01)	0.82 (0.65, 1.04)	0.069
Model 2[b]		1.00 (reference)	0.86 (0.69, 1.07)	0.82 (0.65, 1.02)	0.85 (0.67, 1.09)	0.160
**Distal colon cancer**	214	69	50	57	38	
Unadjusted		1.00 (reference)	0.68 (0.47, 0.97)	0.81 (0.57, 1.15)	0.68 (0.46, 1.01)	0.101
Model 1[a]		1.00 (reference)	0.67 (0.47, 0.97)	0.80 (0.56, 1.14)	0.63 (0.42, 0.94)	0.053
Model 2[b]		1.00 (reference)	0.71 (0.49, 1.03)	0.87 (0.61, 1.26)	0.73 (0.47, 1.12)	0.258
**Rectal cancer**	194	66	49	50	29	
Unadjusted		1.00 (reference)	0.69 (0.48, 1.00)	0.74 (0.51, 1.07)	0.54 (0.35, 0.84)	0.008
Model 1[a]		1.00 (reference)	0.73 (0.50, 1.05)	0.80 (0.55, 1.17)	0.58 (0.37, 0.91)	0.032
Model 2[b]		1.00 (reference)	0.77 (0.53, 1.12)	0.89 (0.61, 1.31)	0.69 (0.43, 1.11)	0.205

a: Adjusted for age (years), sex (male, female) and race (white, non-white). b: Adjusted for model 1 plus body mass index (kg/m^2), smoking status (never, current or former), pack-year of smoking, drinking status (no, yes), physical activity level (min/week), aspirin and ibuprofen consumption (no, yes), family history of colorectal cancer (no, yes), history of diverticulitis (no, yes), colorectal polyps (no, yes), colon comorbidities (including ulcerative colitis, Crohn's disease, Gardner's syndrome, or familial polyposis) (no, yes), energy intake from diet (kcal/day), protein intake from diet (g/day), carbohydrate intake from diet (g/day), and fat intake from diet (g/day).

Reference

1. Stubbendorff A, Sonestedt E, Ramne S, Drake I, Hallström E, Ericson U. Development of an EAT-Lancet index and its relation to mortality in a Swedish population. *Am J Clin Nutr* (2022) 115:705–716. doi: 10.1093/ajcn/nqab369
